# An Effective Neutralizing Antibody Against Influenza Virus H1N1 from Human B Cells

**DOI:** 10.1038/s41598-019-40937-4

**Published:** 2019-03-14

**Authors:** Cheng-Chung Lee, Chih-Ya Yang, Li-Ling Lin, Tzu-Ping Ko, Alarng Hsun-Lang Chang, Stanley Shi-Chung Chang, Andrew H.-J. Wang

**Affiliations:** 1 Institute of Biological Chemistry, Academia Sinica, Taipei, Taiwan; 2 Core Facility for Protein Production and X-ray Structural Analysis, Academia Sinica, Taipei, Taiwan; 3 Department of Science and Innovation, Medigen Biotech Corporation, Taipei, Taiwan; 4 Institute of Biotechnology, National Taiwan University, Taipei, Taiwan

## Abstract

Influenza is a contagious acute respiratory disease caused by the influenza virus infection. Hemagglutinin (HA) is an important target in the therapeutic treatment and diagnostic detection of the influenza virus. Influenza A virus encompasses several different HA subtypes with different strains, which are constantly changing. In this study, we identified a fully human H1N1 neutralizing antibody (32D6) via an Epstein-Barr virus-immortalized B cell-based technology. 32D6 specifically neutralizes the clinically isolated H1N1 strains after the 2009 pandemic but not the earlier strains. The epitope was identified through X-ray crystallographic analysis of the 32D6-Fab/HA1 complex structure, which revealed a unique loop conformation located on the top surface of HA. The major region is composed of two peptide segments (residues 172–177 and 206–213), which form an abreast loop conformation. The residue T262 between the two loops forms a conformational epitope for recognition by 32D6. Three water molecules were observed at the interface of HA and the heavy chain, and they may constitute a stabilizing element for the 32D6-HA association. In addition, each 32D6-Fab is likely capable of blocking one HA trimer. This study provides important information on the strain specificity of 32D6 for the therapeutic treatment and detection of viral infection.

## Introduction

Influenza is a contagious acute respiratory disease caused by the influenza virus infection. It causes mild to severe illness, and it can, at times, lead to death^1,2^. Most people who contract influenza will recover in several days to less than two weeks, but some people will develop complications. Annual epidemics result in a high number of hospitalizations, with an estimated 3–5 million severe cases and 250,000–500,000 deaths globally. Young children, adults aged 65 years and older, pregnant women, and people with certain chronic diseases are among those who are at high risk of serious flu complications, which possibly require hospitalization and sometimes result in death^1,2^. Influenza A infection accounts for the majority of hospitalizations, and it is the only type that causes global pandemic outbreaks (https://www.who.int/).

Influenza A viruses are divided into subtypes based on two proteins on the viral surface: the hemagglutinin (HA) and the neuraminidase (NA). There are 18 different hemagglutinin subtypes (H1-H18) and 11 different neuraminidase subtypes (N1-N11)^3^. The HA molecule initiates infection by binding to receptors on specific host cells. The NA possesses receptor destroying activity, cleaving terminal sialic acid residues from cell-surface glycoproteins and gangliosides to release progeny virus from the host cell. Both are important targets for influenza virus therapeutic treatment and diagnostic detection. Influenza viruses are constantly changing in two different ways: antigenic drift and antigenic shift. Antigenic drift is a mechanism for viruses that accumulate mutations within the genes that occur continually over time as the virus replicates. These changes of HA protein allows the virus to escape the pre-existing immunity in the hosts^1^. Antigenic shift is a sudden change in the antigenicity of influenza A virus. Antigenic shift can be the result of a direct jump from an unknown animal strain to humans or a reassortment of two or more influenza viruses within the same cell. It results in a new virus with the HA or the HA-NA combination that has emerged from an animal population so different from the same subtype in humans that most people do not have immunity to the new virus. Such new viruses may cause pandemics^4^. Antigenic drift occurs in all types of influenza viruses. Antigenic shift, however, occurs only in flu A because it infects more than just human. Vaccination is the most effective way to prevent influenza infection. It has moderate efficacy, good safety, and acceptable tolerability. However, vaccines lack cross-protection and exhibit unsatisfactory efficacy in some high-risk populations, including older people, young children and immunocompromised patients. In addition to vaccines, the general treatment and prophylaxis of influenza is limited to the neuraminidase inhibitors oseltamivir (Tamiflu) and zanamivir (Relenza)^5,6^. The confirmed cases of influenza infection can be treated with both zanamivir and oseltamivir, and if administered within 36 to 48 h of the onset of clinical symptoms, both drugs reduce the duration of illness by 1–1.5 days in patients of all ages. Baloxavir marboxil (Xofluza) is a novel selective inhibitor against influenza cap-dependent endonuclease of influenza A and B viruses and has been approved by the FDA in 2018 for the treatment of acute uncomplicated influenza in people 12 years of age and older who have been symptomatic for no more than 48 hours^7^. However, influenza A virus rapidly acquired resistance against drugs by mutating these viral components. During the 2008–2009 season, over 99% of the H1N1 isolates were resistant to oseltamivir in the United States, Japan, and South Africa^8^. In addition, two influenza A (H3N2) viruses carrying an I38T substitution in the polymerase acidic subunit (PA) show the reduced susceptibility to baloxavir^9^. Patients infected with baloxavir-resistant viruses exhibited prolonged virus shedding, and the median time to symptom alleviation was longer in baloxavir recipients infected with viruses bearing these substitutions than in those infected with viruses that lacked these substitutions^7,10^. Because of the emergence of drug resistance, short treatment windows for antiviral drugs, and the unsatisfactory efficacy of currently available vaccines, there is an unmet medical need for influenza therapy.

Most influenza virus-neutralizing antibodies, elicited via vaccination or infection, bind to the globular head of HA and block the interaction between the viral sialic acid receptors and the host cells. Recently, several studies have shown that influenza-neutralizing antibodies targeting conserved epitopes of influenza virus might offer an alternative treatment. Passive immunotherapy using anti-influenza antibodies has the potential to prevent and treat influenza. Ideally, neutralizing antibodies exhibit a potent effect in direct viral neutralization and induce antibody-dependent cellular cytotoxicity, antibody-dependent cellular phagocytosis and complement-dependent cytotoxicity *in vitro* or *in vivo*. In this study, we identified a human anti-H1N1 (influenza A virus A/California/7/2009 NYMCX-179A) neutralizing antibody via an Epstein-Barr virus (EBV)-immortalized B cell-based technology. It interacts specifically with the HA head portion of H1N1 after 2009. We further characterized the mode of binding by analyzing the complex crystal structure of the fragment for antigen-binding (Fab) and the antigen.

## Results

### Generation and verification of 32D6

**Figure 1:**
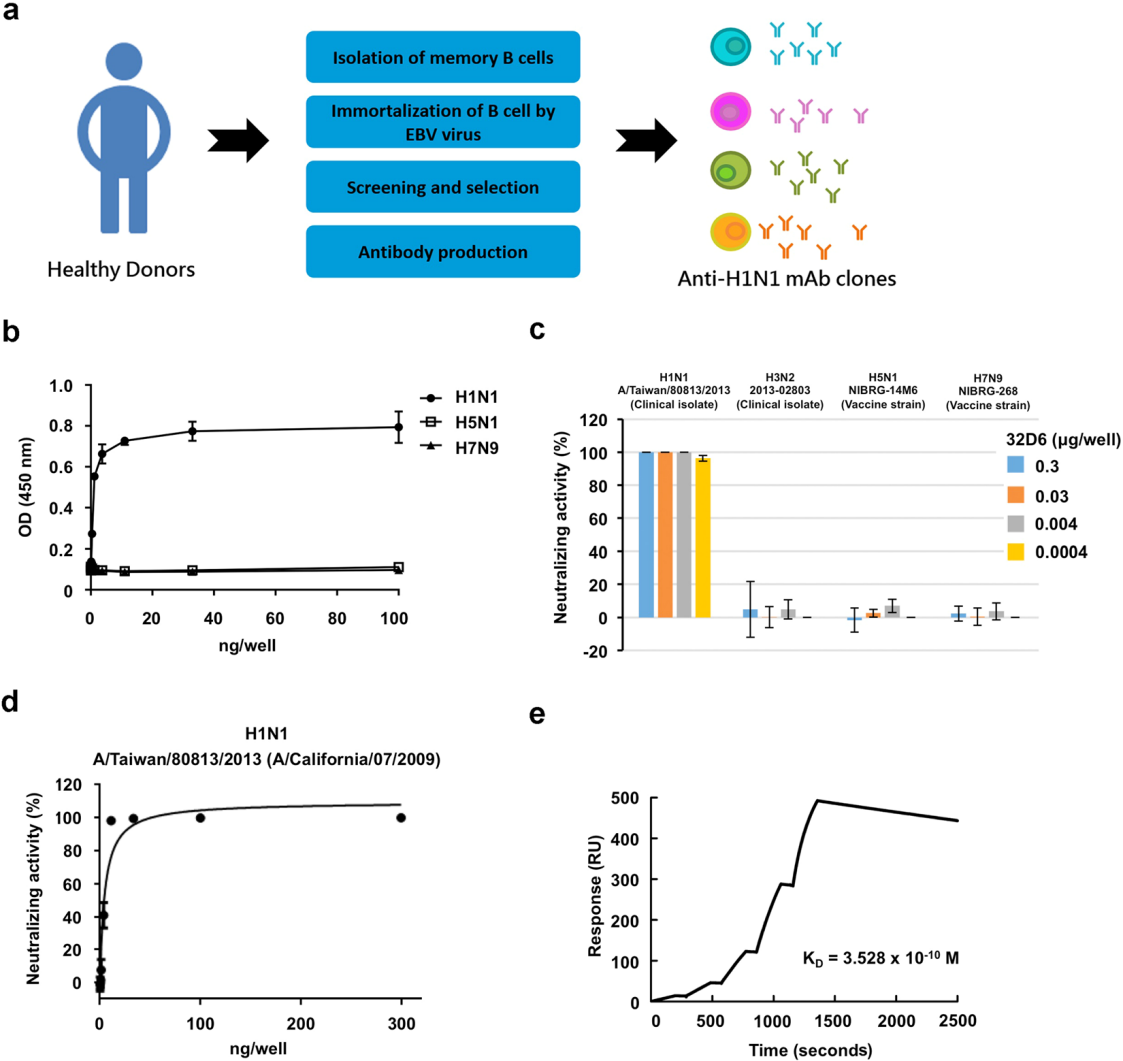
Human monoclonal antibody production and validation. (**a**) Peripheral CD19 + and CD27 + memory B cells were isolated from healthy donors and immortalized by EBV. H1N1 specific clones with binding ability and neutralizing activity were screened and selected via ELISA and neutralization assays. The antibody clone 32D6 was chosen as the lead clone based on its potency. (**b**) 32D6 recognized H1N1, but not H5N1 and H7N9. Inactivated H1N1 (NYMC XA), H5N1 (NIBRG-14M6) and H7N9 (NIBRG-268) viruses were coated on 96-well plates. The binding intensity of serial diluted 32D6 mAbs were examined via ELISA. (**c**) The neutralizing activity of the 32D6 antibody against H1N1 (A/Taiwan/80813/2013), H3N2 (A/Victoria/361/2011), H5N1 (NIBRG-14M6) and H7N9 (NIBRG-268) were examined via a neutralization assay based on a high-content image system. (**d**) The neutralization EC50 value of 32D6 against H1N1 (A/Taiwan/80813/2013) is 0.036 μg/ml. (**e**) The binding kinetics of 32D6 toward HA of H1N1 was measured using the Biacore systems. The K_D_ value is 3.528 × 10^−10^ M.

As described in the experimental section (see below) and as summarized in Fig. 1a, a human monoclonal antibody neutralizing the influenza A virus was obtained from the peripheral blood of vaccinated healthy volunteers using an EBV-based strategy. Peripheral CD19+ and CD27+ memory B cells were isolated from healthy donors and were immortalized via EBV. H1N1-specific clones with binding and neutralizing activities were screened and selected through enzyme-linked immunosorbent assay (ELISA) and neutralization assays. Based on its potency, the antibody clone 32D6 was chosen as the lead clone. It was further characterized using a number of techniques, including ELISA and cell-based neutralization assays, as well as surface plasmon resonance (SPR).

To verify the specificity of the 32D6 monoclonal antibody against the H1N1 virus, additional ELISA was performed by using not only H1N1 (A/Taiwan/80813/2013) but also two other strains, namely, H5N1 and H7N9. As shown in Fig. 1b, the specific binding profile of 32D6 and H1N1 followed a hyperbolic curve, suggesting a strong interaction between the antibody and virus. In contrast, very weak binding was observed for either H5N1 or H7N9 as the binding partner of 32D6. The specificity was also examined via a neutralization assay. As shown in Fig. 1c, 32D6 showed strong neutralization ability against the H1N1 virus. The profile of 32D6 neutralization activity against H1N1 also turned out to be hyperbolic, from which a half maximal effective concentration (EC50) of 0.036 μg/mL was calculated (Fig. 1d). Further, 32D6 showed little activity against all other strains, H3N2, H5N1 and H7N9 (Fig. 1c). In summary, 32D6 is a specific anti-H1N1 monoclonal antibody.

### 32D6 Fab shows high affinity to H1N1 HA1

An intact antibody contains two antigen-binding sites in two Fab. The full-length HA can be divided into two regions, the hemagglutinin head domain (HA1) and hemagglutinin stalk domain (HA2), and it may form a trimer via HA2. Judging by the nature of the divalent antibody and the trimeric HA, and because the HA variations of different strains occur mainly in the HA1 domain, which binds to the host receptor, to investigate the kinetic profile of 32D6 binding to HA, the His-tagged Fab of 32D6 (with the C_H_1 domain) and HA1 of A/California/07/2009 (residues 63–290) were each expressed in human Expi293F cells and were purified. Subsequently, using a Biacore T-200 system, the affinity of 32D6-Fab for the HA1 molecule was determined by measuring its binding kinetics through SPR. Single-cycle kinetics was used for the interaction analysis. 32D6-Fab binds to the HA1 protein with high affinity (Fig. 1e). The dissociation constant (K_D_) value for 32D6-Fab was measured as 3.528 × 10^−10^ M, with an on-rate (*k*_on_) of 2.585 × 10^5^ M^−1^S^−1^ and the off-rate (*k*_off_) of 9.121 × 10^−5^ S^−1^.

### 32D6 recognizes clinically isolated H1N1 strain after 2009

**Figure 2:**
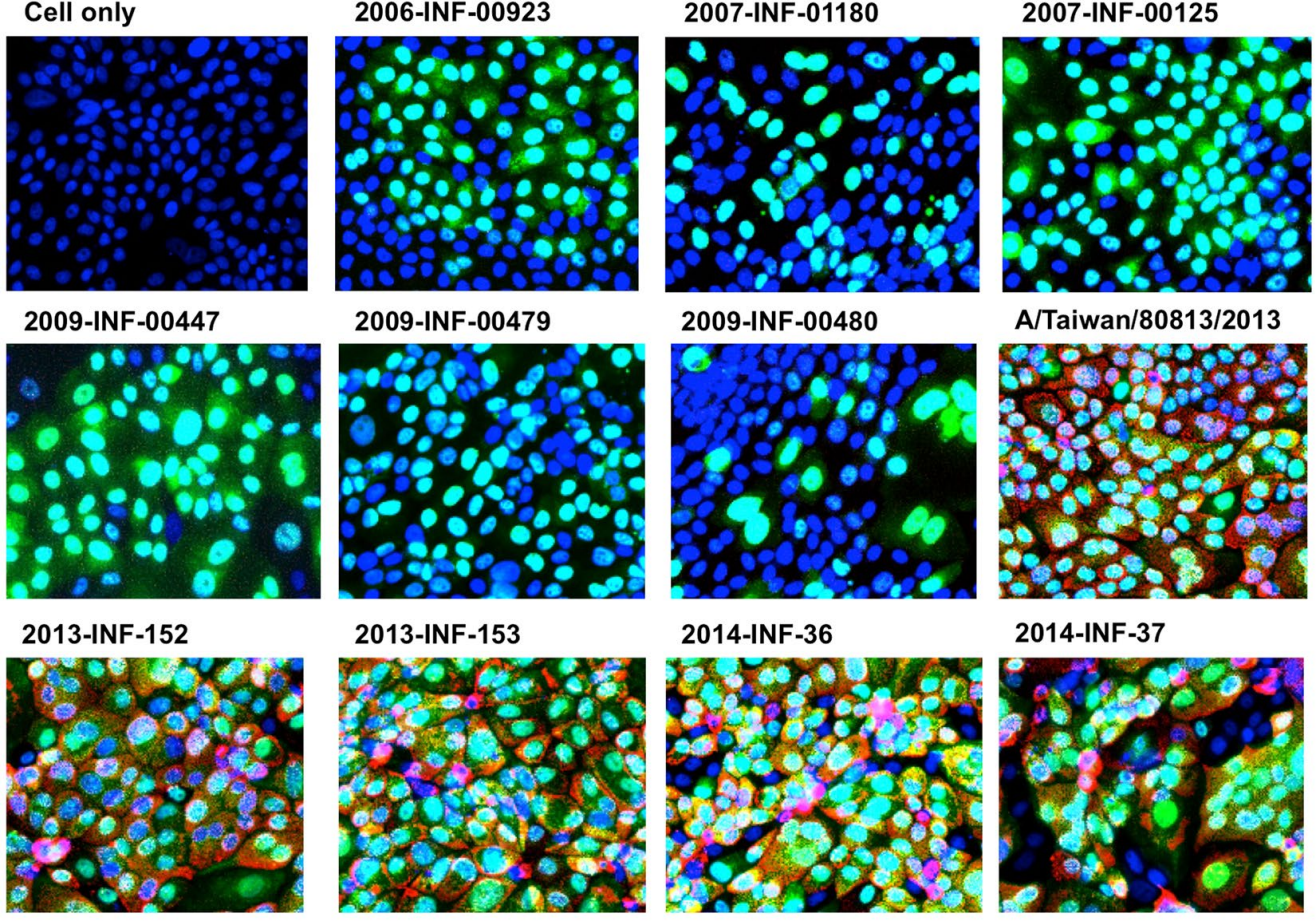
32D6 selectively recognizes H1N1 clinical isolates. MDCK cells were infected with indicated clinical isolated H1N1 influenza virus strains. After 16 hours, infected cells were fixed by 4% paraformaldehyde then double stained by 32D6 (0.3 μg/mL) and FITC conjugated anti-influenza A NP antibody (0.67 μg/mL), followed by Alexa Fluor 647-conjugated mouse anti-human IgG. Nuclei were counterstained by DAPI (2 μg/mL). Signals were detected by high-content analysis system (10x). Blue = DAPI (nucleus); Green = anti-influenza A NP protein Ab-FITC; Red = 32D6 + mouse anti-human IgG-Fluor647. The genotype of each clinical isolate was listed in Table 1.

Influenza A viruses usually underwent recombination and mutation after each pandemic occurrence, producing new strains with varied antigenic determinants. Because the original antigen of 32D6 was A/California/7/2009, several different clinical isolates of H1N1 strains before and after the 2009 pandemic were subsequently tested for neutralization activity. As shown in Fig. 2, six clinical isolates before the pandemic all failed to be recognized by 32D6, whereas five isolates after the pandemic could all be neutralized by 32D6. The corresponding EC50 are listed in Table 1. The EC50 of 32D6 (0.036 μg/mL) for strain A/Taiwan/80813/2013 is slightly higher than those for the four strains of A/California/7/2009, which range from 0.005 to 0.014 μg/mL. Presumably, the isolates of the A/California/7/2009 strain are more closely related to one other (as well as 32D6) than A/Taiwan/80813/2013. Nevertheless, the distinct difference from the six isolates before the 2009 pandemic is very clear.

**Table 1:** Neutralization EC50 of 32D6 against H1N1 clinical isolates before or after the 2009 pandemic.

Clinical isolates before 2009 pandemic	Genotype	EC50 (μg/mL)
2006-INF-00923	A/Solomon Islands/3/2006	>50
2007-INF-01180	A/New Caledonia/20/99	>50
2007-INF-00125	A/Brisbane/59/2007	>50
2009-INF-00447	A/Brisbane/59/2007	>50
2009-INF-00479	A/Brisbane/59/2007	>50
2009-INF-00480	A/Brisbane/59/2007	>50
**Clinical isolates after 2009 pandemic**		
A/Taiwan/80813/2013	A/California/07/2009	0.036
2013-INF-152	A/California/07/2009	0.014
2013-INF-153	A/California/07/2009	0.005
2014-INF-36	A/California/07/2009	0.011
2014-INF-37	A/California/07/2009	0.012

EC50 > 50 μg/mL indicates no neutralizing activity.

### Crystal structure of the 32D6-Fab/HA1 complex

**Figure 3:**
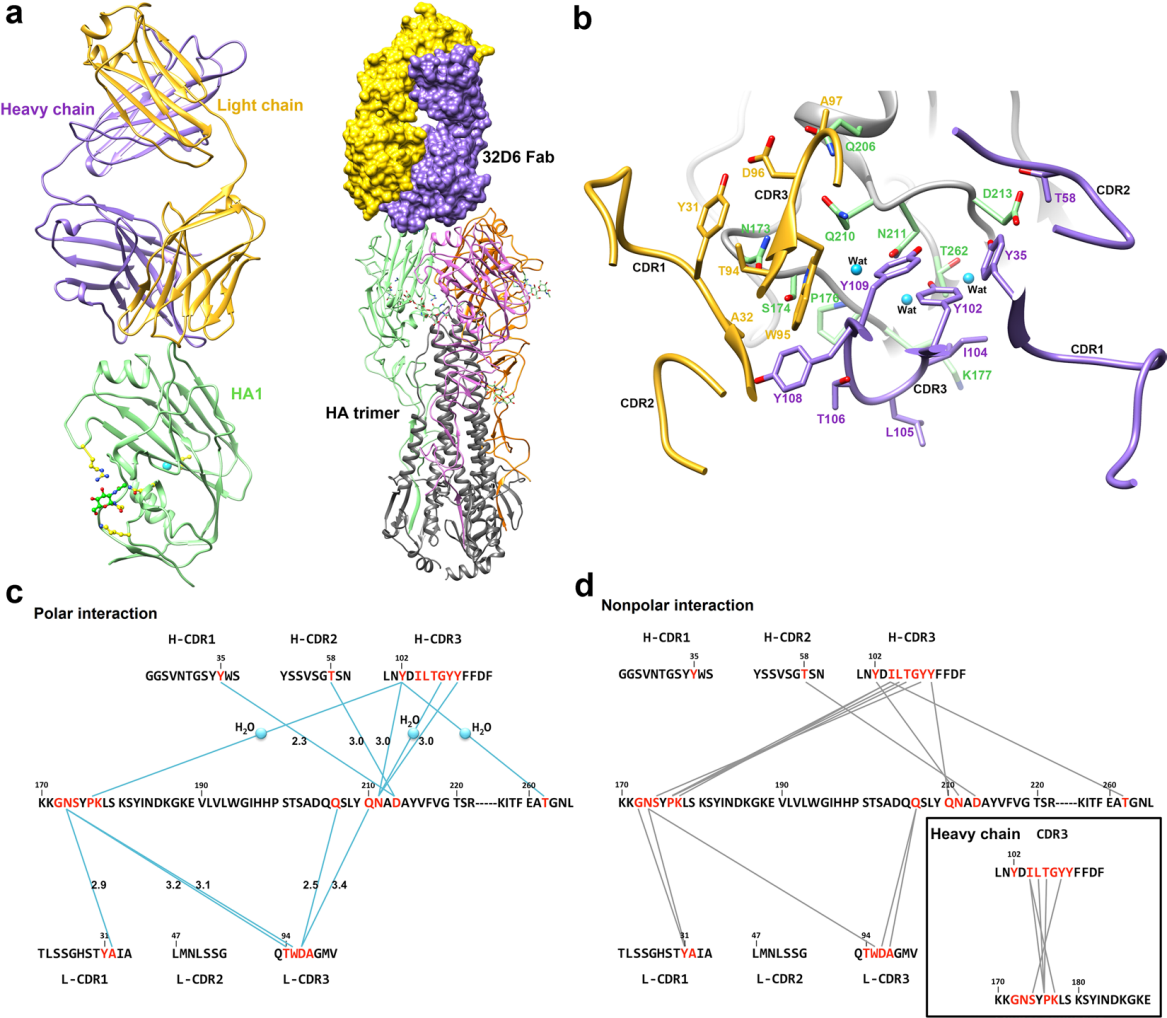
Crystal structure of 32D6-Fab/HA1 complex. (**a**) 32D6-Fab/HA1 complex. The HA1 domain is represented as a green-colored ribbon, and the N-linked oligosaccharide at Asn104 is shown as green sticks. The heavy and light chains are shown in purple and yellow, respectively. The 32D6-Fab/HA1 complex structure is superimposed on the HA trimer (PDB 3LZG). The Fab on the top of the HA trimer is shown in purple and yellow surface for the heavy chain and the light chain, respectively. The trimeric structure of HA with the glycan (green sphere) is shown as a ribbon. Three different HA1 domains from the HA trimer are colored green, orange, and pink, respectively, and all HA2 domains are colored in gray. (**b**) HA1-32D6 interaction interface. The HA molecule is shown as a gray ribbon, and the epitope on HA is shown as stick models with green C atoms. The heavy-chain and light-chain CDRs are shown as purple and yellow ribbons, respectively. The residues involved in contact with HA from CDRs are indicated and are shown as sticks. Three water molecules that mediate the interaction are shown as cyan spheres. (**c**,**d**) Polar interactions and nonpolar interactions between HA1 and 32D6-Fab. The amino acid residues from each CDR and part of the HA sequence from H1N1 are shown. The interaction residues are colored in red, and each interaction is shown as a straight line. The inset shows the nonpolar interaction between the heavy chain CDR3 and the HA1. Three water molecules that mediate the polar interactions are also indicated.

To understand the mechanism by which the antibody 32D6 is capable of neutralizing a large number of H1 viruses, the above 32D6-Fab and HA1 were cocrystallized as a complex, and the structure was determined at 3.15-Å resolution (Table 2). The crystals belong to space group *P*3_1_12 with four 32D6-Fab/HA1 complexes packed into an asymmetric unit (Fig. S1). In the complex structure, one Fab is bound to the top of each globular HA1 domain (Fig. 3a). The model of the 32D6-Fab comprises the heavy chain (residues 1–230) and the light chain (residues 2–217). Together, they bind to the HA1 domain (residues 63–290). 32D6-Fab displays a typical immunoglobulin fold. The complementarity-determining regions (CDRs) are well defined^11^; the light-chain CDR loops are composed of residues 23–34 (CDR-L1), 47–53 (CDR-L2) and 93–100 (CDR-L3), and the CDRs of the heavy chain are composed of residues 26–37 (CDR-H1), 52–60 (CDR-H2) and 100–113 (CDR-H3) (Fig. 3b).

**Table 2:** Data collection and refinement statistics.

	HA1/32D6-Fab
**Data collection**	
Wavelength (Å)	1.0
Space group	*P*3_1_12
Cell dimensions (Å°)	*a* = *b* = 181.7, *c* = 248.1,
	*α* = *β* = 90, *γ* = 120
Resolution (Å)	20–3.15 (3.26–3.15)
Unique reflections	79,824
*R*_merge_ (%)	7.7 (55.4)
*I*/σ(*I*)	14.7 (2.1)
Completeness	98.6 (99.3)
Redundancy	3.2 (3.2)
**Refinement**	
Resolution (Å)	20–3.15
No. of reflections *R*_work_/*R*_free_	69,005/3,824
*R*_work_/*R*_free_	17.4/23.4
**No. of atoms/Avg B factor (Å** ^**2**^ **)**	
Protein	20,364/85.1
Glycan	56/117.1
Others	208/45.1
**RMSD**	
Bond lengths (Å) /Bond angles (°)	0.01/1.53
**Ramachandran statistics (%)** ^**b**^	
Favored	90.22
Outliers	1.62

^a^Values corresponding to the highest-resolution shells are shown in parentheses.

^b^Stereochemistry of the model was validated using MolProbity.

An influenza hemagglutinin contains 566 residues, with a transmembrane segment from residues 531 to 553 and six N-linked glycosylation sites at residues 28, 40, 104, 304, 498 and 557, as predicted by both the *TMHMM (*http://www.cbs.dtu.dk/services/TMHMM) and *NetGlyc* programs (http://www.cbs.dtu.dk/services/NetNGlyc). The HA1 domain contains three predicted glycosylation sites: residues 28, 40 and 104. In the 32D6-Fab complex structure, the glycosylated N104 with a monosaccharide is observed on a SDNGT sequon to stabilize the protein structure of HA1 domain, along with the glycan contact with the side chains of K71, N81, and R238 (Fig. S2)^12^. Glycosylation at the residues N28 and N40 remain uncertain, whereas the other glycosylated residues 304, 498 and 557 were not included in the HA1 in this complex crystal. Notably, a calcium ion is bound to T249 in each HA1 (Fig. S2). The sites of glycosylation and calcium binding are separated from one another on the lateral surface of the HA1 molecule. Neither of them is likely to be involved in the interactions with the Fab.

Based on the HA1 domain, superimposition of this complex onto an HA trimer model indicates that the 32D6-Fab is not blocked from binding to the HA1 domain in the trimer via the same mode as observed in the crystal structure (Fig. 3a). No steric hindrance is observed between the Fab and the other two HA1 domains. The interatomic distances between the Fab region and the other HA1 domains are all greater than 5 Å, and there appears to be no additional Fab-HA1 interaction of significance. However, again with steric hindrance consideration, if any other HA1 in an HA trimer is bound to another Fab, this second Fab would clash severely with the first one. Consequently, via the observed mode of binding, each HA trimer is likely to bind to only one Fab molecule using only one HA1 domain.

### Analysis of the 32D6-Fab/HA1 binding mode

The 32D6-binding site displays a conformational epitope located on the top surface of HA. The major region is composed of two discontinuous linear peptides (residues 172 to 177 and residues 206 to 213) form an abreast loop conformation, and the residues T262 between two loops to form a conformational epitope for 32D6 recognition. The paratope on 32D6 consists of all three heavy-chain CDRs and two light-chain CDRs (L-CDR1 and L-CDR3). L-CDR2 shows no interaction with the antigen. The heavy chain and the light chain have surface areas of 590 Å^2^ buried upon the complex formation.

Figure 3b shows the interface interactions between 32D6-Fab and the influenza virus HA. The side-chain hydroxyl groups from three tyrosine residues (Y35, Y102 and Y109) and the side-chain oxygen atom from T58 in the heavy-chain CDRs provide strong hydrogen bonds to interact with the residues N211, D213 and T262 on the HA molecule. Three water molecules were observed at the interface of HA and the heavy chain. They are consistently observed in all four complexes and show lower *B* values (41.4 Å^2^) than average. Two of these water molecules are hydrogen-bonded to the Y102 side chain from the heavy chain and form hydrogen bonds with P176 and T262 on HA. Another water molecule was used to mediate the interaction between G107 in the heavy-chain CDR3 and N211 on HA. These water-mediated bonds provide important stabilizing force for the intermolecular interaction between the two proteins. The residues T58, Y102, I104, L105, T106, Y108 and Y109 in the heavy-chain CDRs exhibit nonpolar interaction with residues S174, P176, K177, Q210, N211, D213 and T262 of HA (Fig. 3c,d).

In light-chain CDRs, the residues A32, T94, W95, and D96 are hydrogen bonded to the residues N173, Q206 and Q210 in HA. The residues Y31, W95, D96 and A97 have hydrophobic contact with G172, N173 and Q206 in HA (Fig. 3c,d).

## Discussion

As a major surface protein of the influenza virus, HA is involved in receptor binding and facilitates viral entry into the host cell. The conserved receptor-binding site (RBS), which recognizes sialic acid on the host surface glycan, is located in the globular head domain of HA^13^. Subsequent conformational changes in the stem part of HA lead to membrane fusion of the virus and the host cell and the release of the viral content^14^. Influenza virus infection or vaccination can induce a robust antibody response^15,16^. Two types of antibodies have been identified: neutralizing and non-neutralizing antibodies. Conventional neutralizing antibodies bind to the globular head of HA and are often strain-specific. Certain other neutralizing antibodies bind to the more conserved stem region of HA and provide the broadly neutralizing activity to many different strains of flu viruses^17–19^. In general, anti-HA stem antibodies are less effective in viral neutralization, partly because the stem of HA is not as solvent-exposed for binding as the head. However, they can mediate non-neutralizing functions through antibody-dependent cellular-mediated cytotoxicity (ADCC) to eliminate infected cells^16,20,21^. A number of broadly protective monoclonal antibodies against influenza viruses have been isolated and characterized to provide the information for vaccine design or be used as therapeutics^20^.

The antibody 32D6 that we obtained and characterized here is specific for the H1N1 strain of A/California/7/2009. The high specificity is reflected in the results of ELISA and cell-based assays. The affinity of 32D6 to HA, as measured using Biacore, is also strong, consistent with the identical binding mode of four independent 32D6-Fab/HA1 complexes that were observed in the crystal. Interestingly, unlike most other antibodies, 32D6 binds to HA without involving the RBS (Fig. S3a). The 32D6 interface is adjacent to the triad axis in an HA trimer, which cannot bind to additional copies of 32D6 (Fig. S3c). Therefore, the stoichiometry of 32D6-Fab to HA binding is 1 to 3, in contrast to the 1:1 ratio of most other known Fab-HA complexes (Fig. S3b). The HA-head-specific antibody S40 binds to the monomeric state of HA1 (S3cd)^22^. Although the RBS is not blocked directly by 32D6, with the bulky immunoglobulin attached to the top surface of HA trimer, it can no longer bind to the cell-surface receptor. Furthermore, at high antibody concentrations or high 32D6:HA ratios, each HA may tend to bind to one Fab. In this case, the HA trimer would be disrupted and, thus, unable to interact properly with the host receptor.

While many other HA-neutralizing antibodies target the RBS by mimicking host sialoglycan receptors^23,24^, 32D6 uses a different strategy in interacting with HA. The buried surface areas in the 32D6-HA complex formation (590 Å^2^ on the Fab) are not larger than the other known Fab-HA1 complexes (e.g., 640 Å^2^ in PDB 4M5Z); however, because of the contemporaneous presence of several weak bonds between 32D6-Fab and HA1, a stable complex is formed. The interactions include not only direct hydrogen bonds and hydrophobic contacts but also several water-mediated hydrogen bonds. As an all-about medium, water molecules can indeed easily form strong hydrogen bonds. Another feature of particular note is the presence of three phenolic groups of tyrosine residues at the interface. In addition to the hydrogen bonds formed by the hydroxyl groups, the aromatic side chain of tyrosine in the heavy-chain CDRs also has hydrophobic interactions with nonpolar residues on HA.

**Figure 4:**
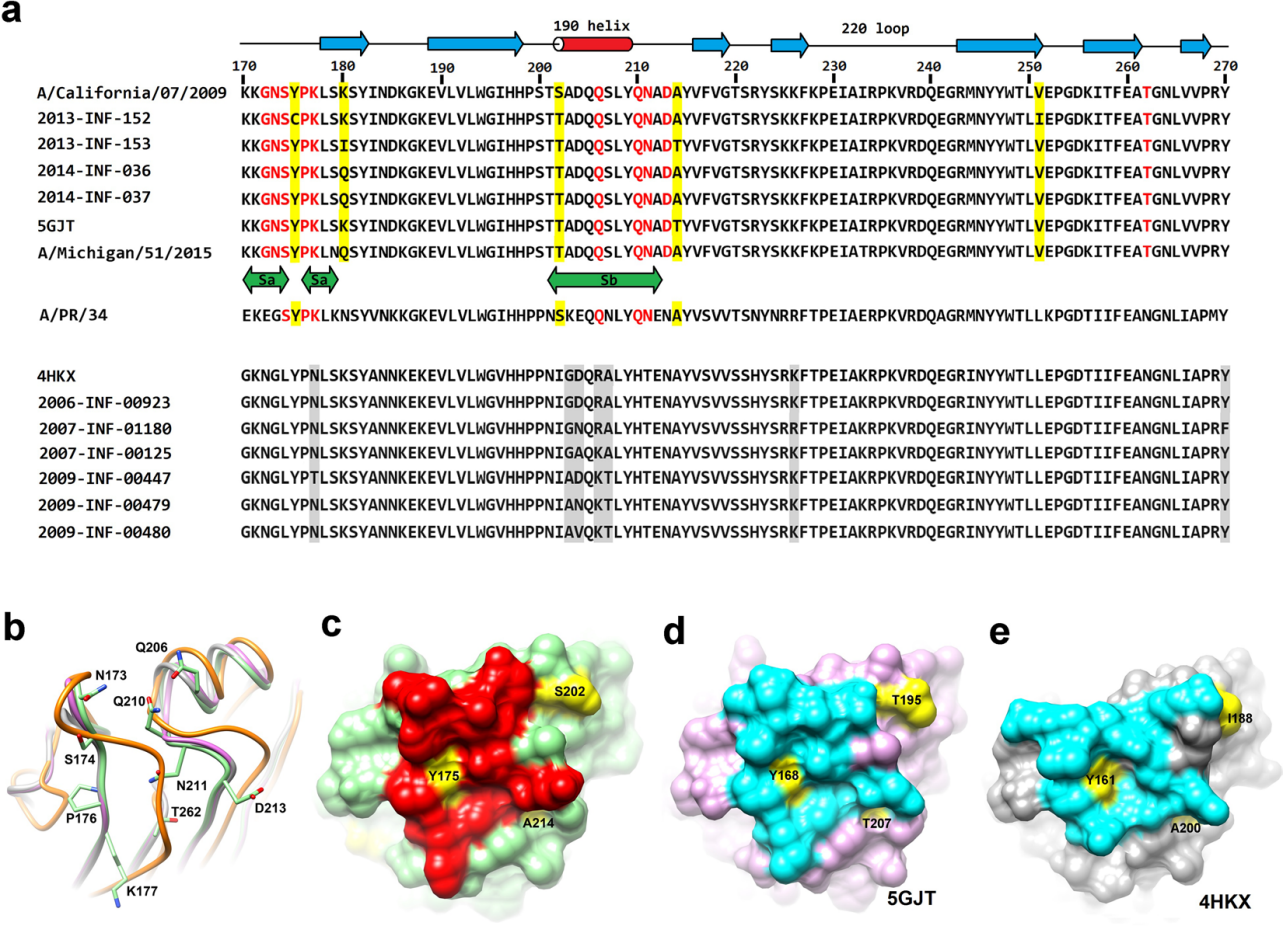
Structure comparison of the 32D6-Fab binding region. (**a**) In the upper part, some sequences of the influenza strains after the 2009 pandemic corresponding to those in Table 1 are aligned. The recent H1N1 strain A/Michigan/51/2015 is included, as well as some earlier strains such as A/PR8/34 in the lower part. Secondary structural elements are shown on the top. The locations of the epitopes Sa and Sb are indicated in the middle. The residue numbers correspond to those of HA in this study. The identified epitope residues are colored in red. Nonconserved residues located on the epitope surface region are marked by a yellow shade. Diverse variable residues in the earlier strain are shaded in gray. (**b**) The epitope-bearing loop conformations of our structure (colored green) are similar to those of PDB 5GJT (pink) and 4HKX (gray). In comparison, the corresponding loops of PDB 4FQK from another influenza species B are longer. (**c**) The epitope region on HA in this study is shown as a surface representation with green background. The identified epitope residues are colored red, and the nonconserved residues in this region are shown in yellow. (**d**,**e**) The epitope residues in PDB 5GJT (pink background) and 4HKX (gray) are colored cyan, with the nonconserved residues in yellow.

A sequence alignment in the epitope region of HA1 is shown in Fig. 4a. The secondary structures and residue numbers are based on the strain in this study. The sequences of a recent strain A/Michigan/51/2015, an older strain A/PR8/34, and two PDB models 5GJT and 4HKX are also included^25,26^. The loop structures are superimposed in Fig. 4b. Our model is colored green, 5GJT (A/California/07/2009) pink, 4HKX (A/Solomon Islands/3/2006) gray, and 4FQK^27^ (B/Brisbane/60/2008) orange. Notably, the loops of 4FQK are much longer than the other three.

The 32D6 antibody can bind to two identified antigenic sites Sa and Sb (Fig. 4a), which are located near the spike tip^28^; the epitope includes the residues G172, N173, S174, P176 and K177 in the Sa site, and the residues Q206, Q210 and N211 in the Sb site. However, no difference in amino acids is found in these regions in our clinical isolates after the 2009 pandemic and the recent A/Michigan/51/2015 H1N1 strain, the conserved epitope can serve as a template for 32D6 neutralizing. The other potential antigenic sites, Ca1, Ca2 and Cb, are not included in the 32D6-binding epitope. Six clinical isolates of the H1N1 virus before the pandemic, all of which failed to be recognized by 32D6, have substantial amino acid change(s) at the Sa and Sb sites. The antibody 2D1, which was isolated from elderly survivors of the 1918 pandemic and which cross-neutralized SC1918 and CA04, exhibits strong binding at the Sa site on H1 HA^29^. The divergence in the antigenic surface of the human H1N1 virus directly correlates with the antibody cross-reactivity.

The HA1/32D6-Fab complex structure clearly elucidates the epitope-CDR interactions. The interface also involves three ordered mediating water molecules, which are similar to another high affinity *herpes simplex virus* neutralization antibody E317^30^. To investigate the relationship between the epitope configuration and the affinity to 32D6, the binding region of HA1 is compared with those of three known structures from PDB 5GJT, 4HKX and 4FQK. The root-mean-square deviations (RMSDs) are 0.52, 0.59 and 1.22 Å for 208, 201 and 87 pairs of the corresponding Cα atoms, respectively. The last complex, 4FQK, is from a different species of influenza B and shows large structural deviations. It is not supposed to react with 32D6.

In contrast, the epitope-containing loop conformations are virtually identical to the other two PDB structures and our structure (Fig. 4b). Nevertheless, sequence comparison of the epitope regions (Sa and Sb) shows clear distinction between 5GJT and 4HKX, each along with the associated strains (Fig. 4a). Mapping of the epitopes onto the 32D6-binding surface of HA1 and the equivalent of 5GJT (Fig. 4c,d) further reveals the similarity of the topology in this region and the identical locations of the conserved key residues that interact with 32D6-Fab. These features are shared by the postpandemic strains but are not found in the prepandemic strains, including 4HKX (Fig. 4e).

Because of the high mutation rate of the influenza virus, annual or seasonal reformulation of vaccines is necessary to confer effective protection. The underlying reason is that the mutations can alter the surface epitopes recognized by antibodies, which then lose the ability to bind strongly to the viruses. In this study, we isolated the human antibody 32D6, which is specific to the H1N1 influenza virus A/California/7/2009, and showed that 32D6 is effective in neutralizing several different isolates after the 2009 pandemic (also known as swine flu). By combining the biochemical and biophysical data with the X-ray crystallographic analysis, a good structural basis is laid down for the strain specificity of 32D6. It turned out that some mutations on the epitope surface do not affect the 32D6 binding. The finding that these mutations cannot escape the antibody binding and the elucidation of the specific epitope-CDR interactions should be useful for the detection and treatment of influenza virus infection.

## Methods

### Cell lines and viruses

The B-lymphoblastoid cell line B95–8 and 293 T cells were cultured in RPMI 1640 medium containing L-glutamine (Invitrogen) supplemented with 10% fetal calf serum and penicillin-streptomycin at 37 °C in a humidified incubator with 5% CO2. Madin-darby canine kidney (MDCK) cells were cultured in dulbecco’s modified eagle medium (DMEM) medium (Invitrogen) supplemented with 10% fetal calf serum. *Spodoptera frugiperda* (Sf9) cells were maintained in Sf-900 II SFM (Invitrogen).

EBV for immortalizing human B lymphocytes was prepared from B95–8 cell line, as described^31^. B95-8 is EBV-producing marmoset B cell line and used as a source of EBV for transformation of human B cells. The culture supernatant of B95-8 was collected and filtered with 0.45 μm sterile syringe filters (Millipore) and stored at −80 °C before use. The vaccine strain of H1N1 virus (NIBRG-122), H5N1 virus (NIBRG-14) and H7N9 virus (NIBRG-268) were obtained from Medigen Vaccine Biologics Co. (MVC, Taiwan). Other influenza virus clinical strains were provided by Taiwan CDC and were used for the recognition assay and neutralization assay. Propagation of those influenza virus clinical strains and determination of the 50% tissue culture infectious dose were performed in MDCK cells according to the Reed-Muench method.

### Purification of human memory B cells and EBV immortalization

All experiments were performed in accordance with IRB guidelines and regulation. B cells were isolated from pre-screened healthy donors (following informed consent and under IRB approved protocols, IRB number: 2013-04-034B #1, Taipei Veterans General Hospital) with high anti-H1N1 virus titer using the combination of Ficoll density gradient centrifugation (GE Healthcare Life Sciences), negative B cell isolation kit (Miltenyi Biotec) and CD27-positive selection kit (Miltenyi Biotec), following the manufacturer’s protocol^32,33^. Memory B cells were then infected with EBV virus according to the protocol^34^. Briefly, memory B cells were seeded in 6-well plates with feeder cells (cobalt-60 gamma-irradiated allogeneic mononuclear cells) in complete medium containing 2.5 µg/mL CpG 2006 (InvivoGen), 400 ng/mL cyclosporine A (Sigma-Aldrich) and 30% EBV supernatant. After 28 days, the culture supernatants were screened for antibodies specific to H1N1 HA from the 2009 pandemic. The full-length cDNA of the desired IgGs were cloned from identified B cell clones for further antibody expression.

### Enzyme-linked immunosorbent assay (ELISA)

ELISAs were performed to examine the H1N1-virus- or HA-binding activities of sera, culture supernatant and purified antibodies. Briefly, inactivated whole H1N1, H5N1 and H7N9 virus were coated on a 96-well plate (Maxisorb, Nunc) at 4 °C overnight followed by blocking with 1% bovine serum albumin (BSA)/PBS (Invitrogen) at 37 °C for 2 h. The serial diluted samples were incubated in wells at 37 °C for 2 h. After a complete wash, the horseradish-peroxidase-labeled anti-human IgG (dilution of 1∶5000) was added and incubated at 37 °C for 1 h. 3,3′,5,5′-tetramethylbenzidine/H_2_O_2_ was added to develop color, and the reaction was stopped with 50 µl of H_2_SO_4_. The amount of chromogen produced was measured based on absorbance at 410 nm and 630 nm using an ELISA reader (SpectraMax, MD, US).

### Neutralization assays, activity determined via high-content image system

Neutralization assays of sera or antibodies were performed as described by a protocol available from the World Health Organization (WHO) with minor modifications^35–37^. Briefly, sera, cell culture supernatants, or purified 32D6 human monoclonal antibodies were serially diluted with DMEM containing 1% BSA and incubated with 100 TCID_50_ (50% tissue culture infectious doses) of H1N1 (A/Taiwan/80813/2013), H3N2 (2013–02803, clinical isolated), H5N1 (NIBRG-14M6) and H7N9 (NIBRG-268) at 37 °C for 2 h. Then the mixture was added into TPCK-trypsin (2 *μ*g/mL) pretreated MDCK cells (1x10^4^ cells per well in 384-well plates) and incubated at 37 °C for 60 min. In the following, the MDCK cells were washed with PBS to remove the supernatant containing free antibodies and virus, then replaced with 10% fetal bovine serum DMEM-base complete medium for another 16 h of culture at 37 °C. After that, the MDCK cells were fixed with 4% paraformaldehyde and were double stained with the anti-nucleoprotein (NP)-fluorescein isothiocyanate (FITC)-labeled antibody (Millipore MAB8257F) and 4′,6-diamidino-2-phenylindole (DAPI) (SIGMA-B2261). The infection efficiency was quantified and analyzed using a high-content image system (ImageXpress Micro, Molecular Device, Sunnyvale, CA, USA). The neutralization activity of antibodies was calculated according to the equation: (A–B)/A × 100%, wherein ‘A’ represents the cell counts of NP/DAPI-double positive cells in virus infection control, and ‘B’ represents the cell counts of NP/DAPI-double positive cells in the testing sample.

### Preparation of the 32D6-Fab and HA1 proteins

The cDNA sequence corresponding to the HA1 domain (residues 63–290) of HA from A/California/07/2009 (H1N1; Accession No. ACP41953.1) with the C-terminal His-tag was constructed into the pcDNA 3.4 vector (A14697, Invitrogen) for protein expression. The HA1 domain was expressed in Expi293F cells (A14527, Gibco) with transient transfection using the ExpiFectamine 293 transfection kit (A14525, Gibco). The culture supernatants were collected for purification using a Ni^2+^-covalent-bound HisTrap excel prepacked column (17-3712-06, GE Healthcare). The His-tagged HA1 proteins were eluted using a solution containing 20 mM sodium phosphate, 500 mM NaCl, and 300 mM imidazole, pH 7.4.

The 32D6-Fab expression vector was derived from the IgG expression plasmid, pIgG (U.S. patent No. 5736137), which did not contain the C_H_2 and C_H_3 domains of the heavy chain but contained an additional His-tag at the C-terminus of the C_H_1 domain of the heavy chain. The 32D6-Fab was also expressed in Expi293F cells and purified via a HisTrap excel column using a gradient from 10 mM to 500 mM imidazole in solution (20 mM sodium phosphate, 500 mM NaCl, pH 7.4). The fractions containing 32D6-Fab were eluted at 300 mM imidazole.

### Biacore

The single-cycle kinetics of HA1 and 32D6-Fab was measured on Biacore T-200 (GE Healthcare) at 25 °C. 32D6-Fab was immobilized on a CM5 chip (GE Healthcare) at 260 resonance units (RUs) using the standard procedure for amine coupling through the EDC/NHS reaction. Five different analyte concentrations (0.37–30 nM) of HA1 were used in each cycle at a constant flow rate of 65 μL/min in the running buffer, which contained 10 mM HEPES, pH 7.4, and 150 mM NaCl. The surface was regenerated after each cycle. The association was measured for 200 secs, and the final dissociation time was 1200 secs. The kinetic data was analyzed using the Biacore T200 evaluation software.

### Crystallization and data collection

To obtain the 32D6-Fab/HA1 complex, purified 32D6-Fab and HA1 were premixed in a 1:1 molar ratio at 4 °C overnight. The mixture was loaded onto the gel-filtration column (Superdex 200 prep-grade XK16/70, GE Healthcare), and the protein complex was eluted at a flow rate of 0.5 ml/min at 4 °C in a buffer solution consisting of 50 mM Tris and 100 mM NaCl, pH 8.0. The optical absorbance at 280 nm was used to monitor the eluted protein complex.

The HA1/32D6-Fab complex crystals were grown by mixing 1 µl of protein solution with 1 µl of reservoir solution using the sitting-drop vapor-diffusion method at 293 K. The crystals were obtained in a reservoir solution consisting of 14.4% (w/v) polyethylene glycol 8000, 160 mM calcium acetate, 20% (v/v) glycerol, 80 mM sodium cacodylate/hydrochloric acid, pH 6.5. The crystals were flash-cooled, and the diffraction patterns were recorded at cryogenic temperatures. The diffraction data were collected at a wavelength of 1.0 Å on Taiwan Photon Source (TPS) beamline TPS-05A of National Synchrotron Radiation Research Center (NSRRC) in Taiwan using a Rayonix MX300-HS CCD detector. The diffraction data were processed and scaled using HKL-2000^38^.

### Structure determination and refinement

The HA1/32D6-Fab complex crystal structure was determined via molecular replacement using the software *PHASER*^39^ of PHENIX^40^ and the IgG1-Fab (PDB entry 3N9G) and the HA1 (PDB entry 3GBN^41^) fragments as the search models. The HA1/32D6-Fab complex crystals belonged to the space group *P*3_1_12, with four HA1/32D6-Fab complexes in an asymmetric unit. Throughout the refinement using REFMAC5^42^ of the CCP4 suite^43^, a randomly selected 5% of the data were set aside for cross-validation by the *R*_free_ value. Manual modifications of the models were performed using the program Coot^44^. The complex structure was refined to 3.15-Å resolution, from which *R*_work_ and *R*_free_ values of 17.5 and 23.3%, respectively, were obtained.

Data-collection and final model statistics are shown in Table 2. The molecular figures were produced using Chimera^45^. The atomic coordinates and structure factors of the crystal structure of the HA1/32D6-Fab complex have been deposited in the Protein Data Bank with accession code 6A4K.

### Ethics statement

This study was approved by the institutional review board (IRB) of Taipei Veterans General Hospital (IRB number: 2013-04-034B #1). All healthy volunteers provided their written informed consent to participate in this study.

## Supplementary information

An Effective Neutralizing Antibody Against Influenza Virus H1N1 from Human B Cells

## Data Availability

The authors confirm that all relevant data are included in the paper and /or its supplementary information files.
