# Supplementary material for: An Effective Neutralizing Antibody Against Influenza Virus H1N1 from Human B Cells
Source: Sci Rep. 2019 Mar 14;9:4546. doi: 10.1038/s41598-019-40937-4 (PMC6418199; doi:10.1038/s41598-019-40937-4)

An Effective Neutralizing Antibody Against Influenza Virus H1N1 from Human B Cells

Cheng-Chung Lee[1,2], Chih-Ya Yang[3], Li-Ling Lin[1,2], Tzu-Ping Ko[1], Alarng Hsun-Lang Chang[3], Stanley Shi-Chung Chang[3,4]*, Andrew H.-J. Wang[1,2]*

Affiliations

[1]Institute of Biological Chemistry, Academia Sinica, Taipei, Taiwan, [2]Core Facility for Protein Production and X-ray Structural Analysis, Academia Sinica, Taipei, Taiwan, [3]Department of Science and Innovation, Medigen Biotech Corporation, Taipei, Taiwan, [4]Institute of Biotechnology, National Taiwan University, Taipei, Taiwan

Correspondence:

ahjwang@gate.sinica.edu.tw, sscchang@medigen.com.tw

Supplementary Information

Figure S1. (a) An asymmetric unit of the 32D6-Fab/HA1 complex crystal. (b) The four complexes are superimposed on the right, with RMSD varying from 0378 to 0.876 Å for 455 to 612 matched Cα pairs.

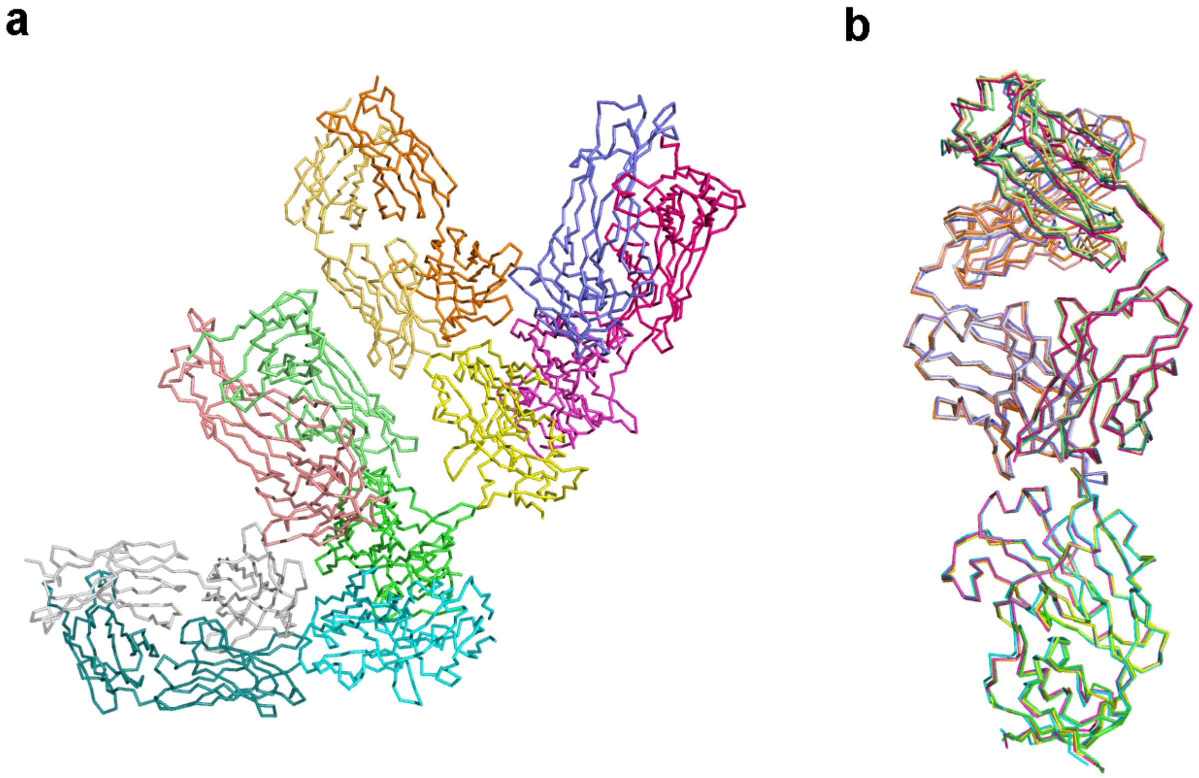

Figure S2. Glycosylation site. The Asn104-linked N-acetylglucosamine (GlcNAc) on HA1 is bound to K71, N81 and R238. A calcium ion bound to T249 of HA1 molecule is also shown.

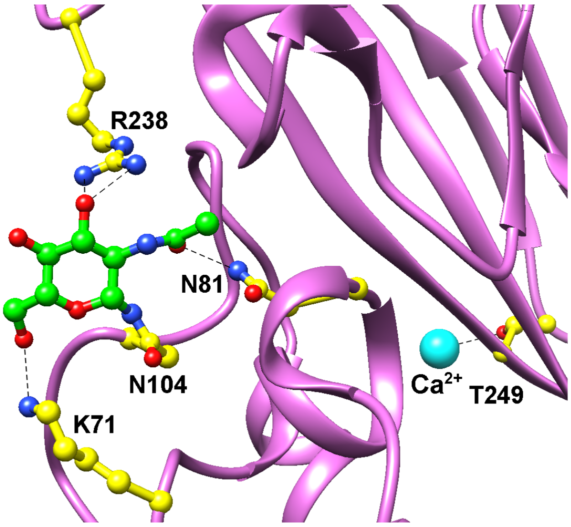

Figure S3. Comparison of Fab-binding sites on HA. (a) The Fab of 32D6 is colored cyan and magenta. The bound HA1 is presented in green. The superimposed yellow model of HA is from PDB 3UBE, with the bound sialoglycoside shown as sticks. (b) Two examples of trimeric Fab-HA1 complexes exhibit a 1:1 ratio. (c) One Fab molecule of 32D6 binds to the HA trimer and compared with the S40 binding site. (d) S40 binds to the HA1 monomer at the trimer contact interface. The binding of S40 inhibits the trimer formation of HA1 molecules.

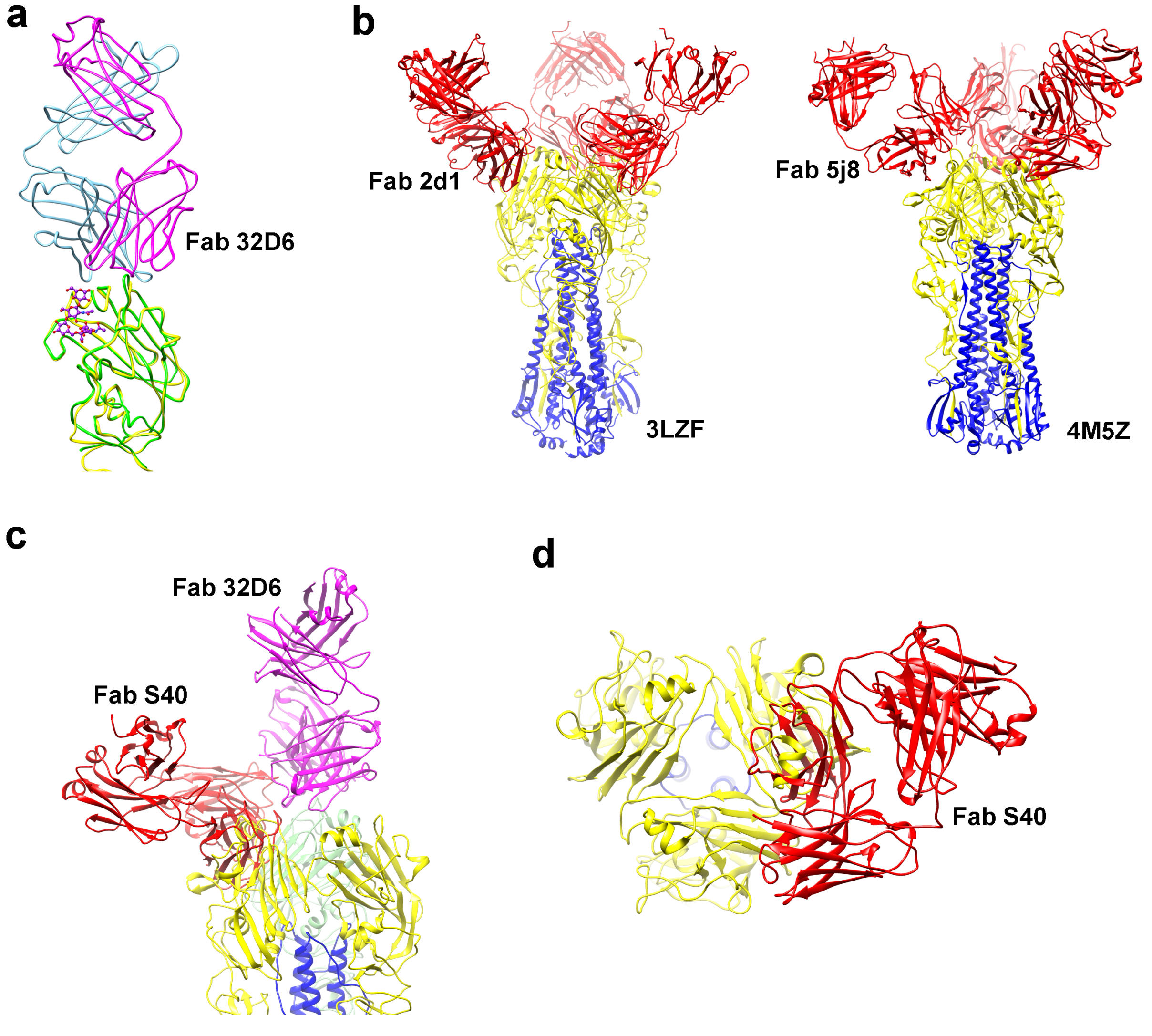

Supplement: Supplementary file 1 — An Effective Neutralizing Antibody Against Influenza Virus H1N1 from Human B Cells [file 41598_2019_40937_MOESM1_ESM.pdf]
